# The Role of Flow Chemistry on the Synthesis of Pyrazoles, Pyrazolines and Pyrazole-Fused Scaffolds

**DOI:** 10.3390/molecules30071582

**Published:** 2025-04-02

**Authors:** Catarina M. Correia, Artur M. S. Silva, Vera L. M. Silva

**Affiliations:** Associated Laboratory for Green Chemistry (LAQV) of the Network of Chemistry and Technology (REQUIMTE), Department of Chemistry, University of Aveiro, 3810-193 Aveiro, Portugal; catarina.m.c@ua.pt

**Keywords:** flow chemistry, green chemistry, synthesis, pyrazoles, pyrazolines, indazoles

## Abstract

Nitrogen-containing heterocycles are fundamental scaffolds in organic chemistry, particularly due to their prevalence in pharmaceuticals, agrochemicals and materials science. Among them, five-membered rings, containing two nitrogen atoms in adjacent positions—such as pyrazoles, pyrazolines and indazoles—are especially significant due to their versatile biological activities and structural properties, which led to the search for greener, faster and more efficient methods for their synthesis. Conventional batch synthesis methods, while effective, often face challenges related to reaction efficiency, scalability and safety. Flow chemistry has emerged as a powerful alternative, offering enhanced control over reaction parameters, improved safety profiles and opportunities for scaling up synthesis processes efficiently. This review explores the impact of flow chemistry on the synthesis of these pivotal heterocycles, highlighting its advantages over the conventional batch methods. Although indazoles have a five-membered ring fused with a benzene ring, they will also be considered in this review due to their biological relevance.

## 1. Introduction

The pyrazole moiety and related scaffolds are well-known in the pharmaceutical and medical fields as agrochemicals, and in materials science due to their structural and electronic properties, and biological activities, which are mainly analgesic, anti-inflammatory, anticancer, antioxidant and antifungal, among others [1,2,3,4]. Due to the increasing interest in this type of compound, a broad range of synthetic methods has been developed and described over the years. In 2023, Hasani and coworkers published a review on methods for the synthesis of pyrazoles [5]. However, the most common methods—batch methods—while effective, present some major issues such as long reaction times; lack of selectivity; and, in some cases, low yield and safety concerns, thus urging researchers to develop safer and more efficient processes [6,7]. Flow chemistry, also known as continuous flow processing, involves performing chemical reactions in a continuously flowing stream rather than in a static batch reactor [8]. Thus, flow chemistry has become a compelling alternative due to its key advantages, which include (i) enhanced reaction control, meaning that a precise control over temperature, pressure and reaction time is possible; (ii) safety, because it allows a better management of exothermic and hazardous reactions; (iii) scalability, facilitating the transition from laboratory to industrial scale; and (iv) efficiency, as it allows a significant reduction in reaction time and improvement in reaction yield [9,10].

These features make flow chemistry particularly suited for synthesizing more complex molecules, such as nitrogen-containing heterocycles. In the next sections, relevant examples of the application of flow chemistry in the synthesis of pyrazoles, pyrazolines and indazoles, reported in the last ten years, will be presented. It is noteworthy that only fifteen publications (research papers and reviews) were found for this period of time, whilst for the flow synthesis of indazoles, only three papers were reported. These numbers show that there is space for further development of research in this area.

## 2. Pyrazoles, Pyrazolines and Fused Scaffolds

**Figure 1:**
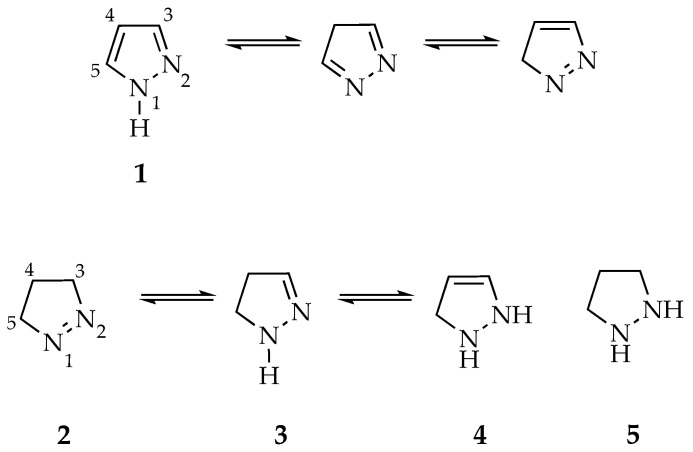
Chemical structures and numbering of pyrazole **1**, dihydropyrazole (pyrazoline) tautomers **2**–**4** and pyrazolidine **5**.

Pyrazoles **1** are a family of aromatic heterocyclic compounds characterized by a five-membered ring constituted by three carbons and two adjacent nitrogen atoms, located at 1- and 2-positions [11,12]. *N*-Unsubstituted pyrazoles often exhibit three identical and inseparable tautomeric forms, owing to rapid interconversion in solution, making it typically challenging to definitively assign the proton resonances of the pyrazole core in proton nuclear magnetic resonance (^1^H NMR) spectra. Three partially reduced forms—1-pyrazoline **2**, 2-pyrazoline **3** and 3-pyrazoline **4**—may also exist, as well as a fully reduced form known as pyrazolidine **5** (Figure 1) [13].

**Figure 2:**
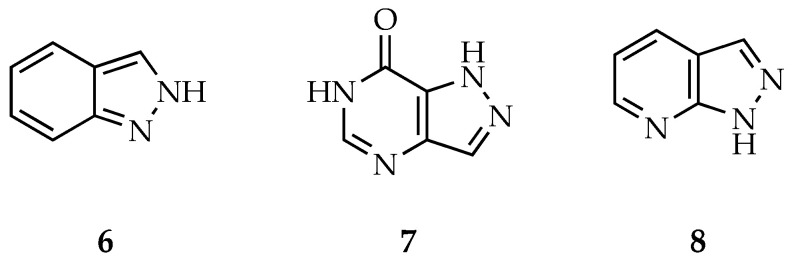
Chemical structures of indazole **6**, pyrazolopyrimidinone **7** and pyrazolopyridine **8** scaffolds.

Additionally, the pyrazole core can be fused with other rings—for example, benzene, pyrimidone and pyridine rings—to form indazole **6**, pyrazolopyrimidinone **7** and pyrazolopyridine **8** scaffolds, respectively (Figure 2). Recent reviews by Mal et al., and Xu and colleagues, gather all the information regarding biological properties and applications of these pyrazole hybrids, highlighting the fact that some commercial drugs already incorporate these scaffolds in their structure [14,15].

The increasing interest that pyrazoles and their related derivatives (pyrazolines and indazoles)—both natural and synthetic analogues—have been receiving over the years is due to their various pharmacological properties such as anticancer, analgesic, anti-inflammatory, antioxidant, antibacterial, antifungal, antipyretic, antidepressant, anticonvulsant, antidiabetic and cannabinoid activities, among others [16,17,18,19,20,21,22,23,24]. In fact, the pyrazole scaffold and its derivatives are present in several known drugs, such as celecoxib (Celebrex^®^), sildenafil (Viagra^®^), rimonabant, lonazolac, fomepizole, penthiopyrad, doramapimod, sulfaphenazole, lonidamine and bendazac. Furthermore, these *N*-heterocycles can easily form bonds with a variety of enzymes and receptors in biological targets due to their ability to form various weak interactions and π-stacking, which is the reason why they are key scaffolds in the field of medicinal chemistry [25]. On the other hand, the electronic properties of pyrazoles and pyrazolines—their intrinsic fluorescence, crystal structures and solid-state properties—make them valuable in various materials science applications such as in fluorescent probes and sensors; optoelectronic, photoluminescent and energetic materials; as organic fluorophores based on small molecules; and as dyes, among other applications [26,27,28,29,30,31].

### 2.1. Synthesis of Pyrazoles and Pyrazolines

**Scheme 1:**
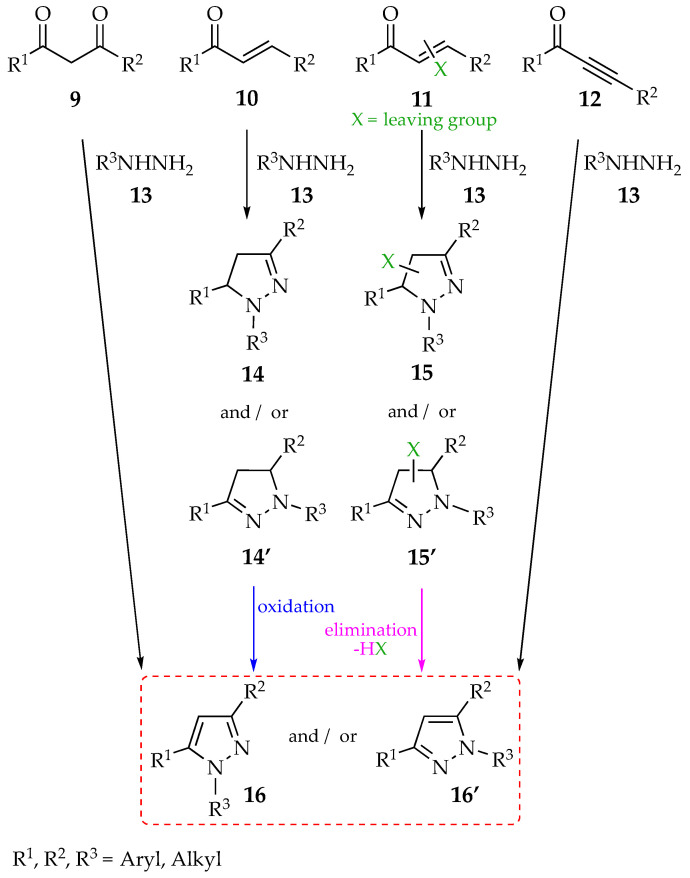
Cyclocondensation of carbonyl compounds **9**–**12** with hydrazine derivatives **13**.

**Scheme 2:**
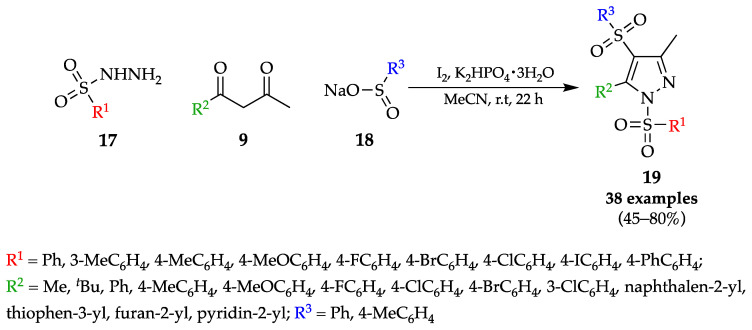
Multicomponent synthesis of pyrazoles.

**Scheme 3:**
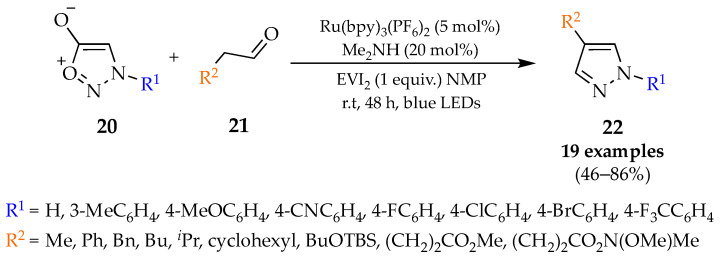
1,3-Dipolar cycloaddition of sydnones with carbonyl compounds.

**Scheme 4:**
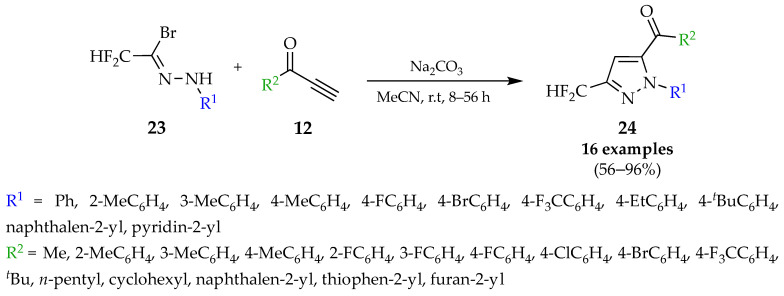
1,3-Dipolar cycloaddition of nitrilimines with terminal alkynes.

**Scheme 5:**
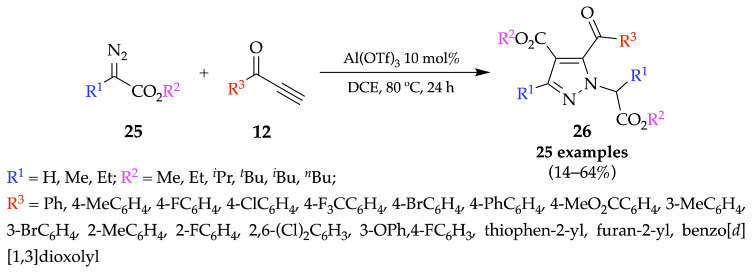
1,3-Dipolar cycloaddition of diazo compounds with terminal alkynes.

Several methods for the synthesis of pyrazoles have been developed and described over the years, with the most employed or classical ones being (a) cyclocondensation of carbonyl compounds **9**–**12** with hydrazine derivatives **13** (Scheme 1); (b) multicomponent reactions (Scheme 2); and (c) 1,3-dipolar cycloadditions of sydnones (Scheme 3), nitrilimines (Scheme 4) or diazo compounds (Scheme 5). These methods were described in detail in a recent review [5,32,33,34,35].

### 2.2. Synthesis of Indazoles

**Scheme 6:**
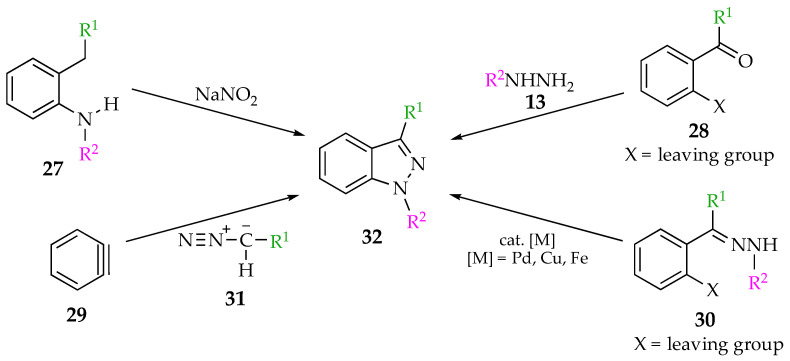
Common strategies for the synthesis of *N*-aryl-1*H*-indazoles **32**.

When it comes to the synthesis of indazoles, the most common strategies are (a) diazotization of *o*-alkyl-substituted anilines **27**, (b) condensation of *o*-substituted carbonyl compounds **28** with hydrazine derivatives **13**, (c) [3 + 2] cyclization of benzynes **29** with diazomethanes **31** and (d) transition-metal-catalyzed intramolecular amination of *o*-haloarylhydrazones **30** or direct C–H amination of arylhydrazones (Scheme 6) [36,37,38].

Although these *N*-heterocyclic compounds have been deeply studied in the last few years, the truth is that most of the synthetic approaches developed to achieve pyrazoles and pyrazole derivatives still require improvements, especially in terms of increases in the yields, regioselectivity and up-scaling. Moreover, the handling of hazardous materials and intermediates imposes a major obstacle, not only due to the risk of exposure but also because it limits the available functional groups and, consequently, the number of derivatives. Thus, it is crucial to develop more efficient and safer methods for the synthesis of pyrazoles and pyrazole derivatives [39]. In this line of thought, flow chemistry has been pointed out as a viable alternative over batch methods.

## 3. Principles of Flow Chemistry

**Figure 3:**
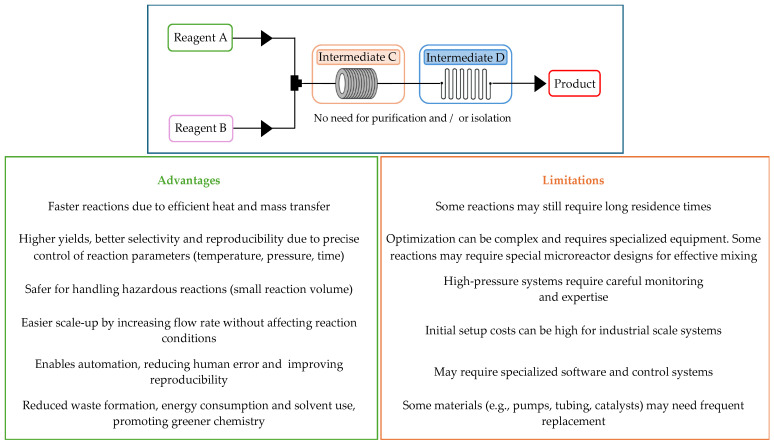
Principles, advantages and limitations of flow chemistry.

The field of flow chemistry has been growing over the last years, due to its major advantages and applications in the context of Green Chemistry towards the development of more sustainable synthetic approaches. This concept is based on a continuous stream of different starting materials, which are introduced by pumps or syringes and mixed in a continuous reactor. Rigorous control of both temperature and pressure, as well as the flow rate of each reagent and/or mixture, is possible when using this methodology, improving reactions’ efficiency and reproducibility [40]. Additionally, enhanced mass and heat transfer, and reduced energy consumption and waste formation, are two other important advantages, making flow chemistry a greener method [41]. Products are usually obtained with higher yield and selectivity when compared to batch conditions, increasing product quality. Another major benefit of reactions under flow conditions is the ability to handle and generate hazardous and/or toxic intermediates in situ, avoiding workups of such compounds and therefore improving environmental protection and operator safety. Furthermore, there is no accumulation of considerable amounts of dangerous compounds since the flow process does not require high reaction volumes (Figure 3). While offering these advantages, flow chemistry also presents technical challenges that need to be addressed for its widespread adoption. For example, achieving uniform mixing in continuous flow systems can sometimes be challenging, especially for reactions that require precise control of reactant distribution, leading to variations in product yield and selectivity. Another problem can be the difficulty in maintaining optimal reaction temperatures for highly exothermic or endothermic reactions. Inadequate heat transfer control can lead to hot spots, thermal degradation or formation of by-products. Also, precipitation of solids, polymerization or deposition of reaction by-products can clog microreactors. Finally, multi-phase, gas–liquid and liquid–solid reactions may be more difficult to handle, requiring specialized reactor designs to ensure efficient contact between phases. Likewise, reactions involving long residence times or specific catalysts may not be well-suited for continuous flow (Figure 3). To overcome these difficulties, tailored reactor designs or hybrid batch–flow approaches may be necessary. Interdisciplinary collaborations between chemists and engineers to surpass these obstacles have contributed to the development of innovative reactors, control systems and process optimization, boosting the development of flow chemistry.

## 4. Flow Chemistry in the Synthesis of Pyrazoles and Related Derivatives

### 4.1. Flow Synthesis of Pyrazoles and Pyrazolines

**Scheme 7:**
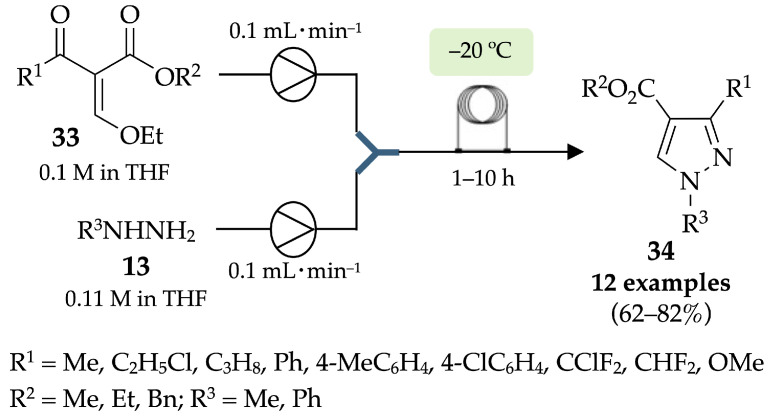
Flow synthesis of pyrazole-4-carboxylate derivatives **34**.

In 2019, Das et al. established a flow setup for the synthesis of pyrazoles **34** from vinylidene keto ester intermediates **33** and hydrazine derivatives **13** [42]. Pyrazole derivatives were achieved in good to very good yields (62–82%) with excellent regioselectivities (95:5, 96:4, 98:2) (Scheme 7).

**Scheme 8:**
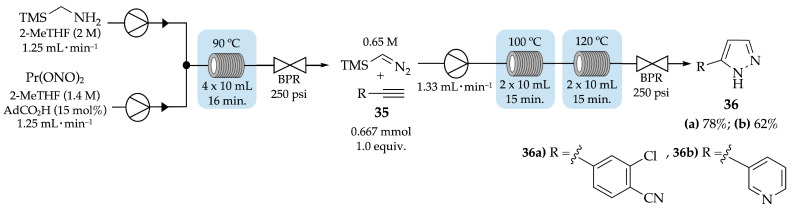
Continuous-flow 1,3-dipolar cycloaddition of alkynes with trimethylsilyldiazomethane to give pyrazoles **36a**,**b**.

Audubert and colleagues have reported the continuous-flow 1,3-dipolar cycloaddition of terminal alkynes **35** with trimethylsilyldiazomethane (TMSCH_2_N_2_) (Scheme 8) [43]. In the first step, the diazotization reaction of trimethylsilylmethylamine (TMSCH_2_NH_2_), under flow conditions, produced trimethylsilyldiazomethane (TMSCH_2_N_2_) in moderate yield (65%), with a short residence time of 16 min. The cycloaddition was performed in continuous flow at 100 °C for 15 min; then at 120 °C for another 15 min with a flow rate of 1.33 mL⋅min^−1^ when using 2-chloro-4-ethynylbenzonitrile **35a** as the terminal alkyne; and at 100 °C for 30 min with the same flow rate for 3-ethynylpyridine **35b**. 2-Chloro-4-(1*H*-pyrazol-5-yl)benzonitrile **36a** and 3-(1*H*-pyrazol-5-yl)pyridine **36b** were obtained with 78% and 62% yields, respectively.

**Scheme 9:**
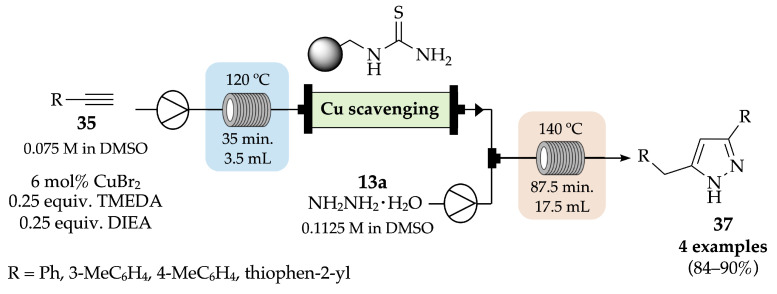
Synthesis of 3,5-disubstituted pyrazoles **37** under flow conditions.

In 2019, a new uninterrupted two-step flow strategy was developed by Ötvös et al., aiming to synthesize 3,5-disubstituted pyrazoles **37** from terminal alkynes **35** and hydrazine monohydrate **13a** [44]. First, a copper-catalyzed homocoupling of the alkyne derivatives led to the in situ formation of the correspondent 1,3-diyne intermediates, which subsequently reacted with hydrazine in a Cope-type hydroamination, giving the expected disubstituted pyrazoles **37** with very good yields (84–90%) (Scheme 9). This methodology not only allows easy access to a diverse set of pyrazoles, without the need to isolate any intermediates, but also decreases the reaction time to around 2 h compared to the 28 h required in batch conditions [45]. Nevertheless, increasing the reaction time up to 16 h, in flow conditions, allowed the scale up of the reaction, achieving 0.52 g of the 3-(thiophen-2-yl)-5-(thiophen-2-ylmethyl)-1*H*-pyrazole derivative with 81% yield.

**Scheme 10:**
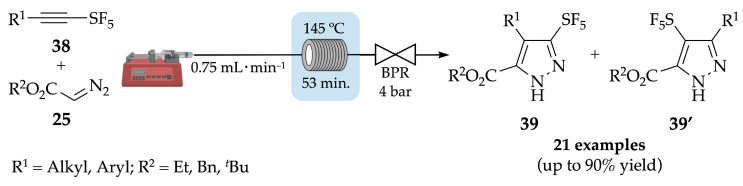
Flow synthesis of pentafluorosulfanylpyrazoles **39** and **39′**.

More recently, Paquin and co-workers have synthesized a new library of 21 pentafluorosulfanylpyrazoles **39** and **39′**, also based on a 1,3-dipolar cycloaddition of SF_5_-alkynes **38** with diazoacetates **25**. Firstly, a preliminary reaction under batch conditions (80 °C, 48 h) gave the expected pyrazole with a moderate 51% yield as a mixture of regioisomers (65:35). To improve the yield, decrease the reaction time and minimize the risks associated with the heating of diazo compounds, a flow approach was developed. As a representative example, a mixture of the ethynylpentafluoro-λ^6^-sulfane derivatives **38** with the diazo counterpart **25** was injected through a PFA reactor at 145 °C for 53 min (Scheme 10). After purification, the products were obtained with up to 90% yield, as a mixture of isomers (with up to 73:27 ratio) [46]. It is noteworthy to mention that 3-SF_5_-pyrazoles **39** were obtained as major isomers when alkyl-substituted SF_5_-alkynes were used, while 4-SF_5_-pyrazoles **39′** were achieved as major isomers when using aryl-substituted SF_5_-alkynes.

**Scheme 11:**
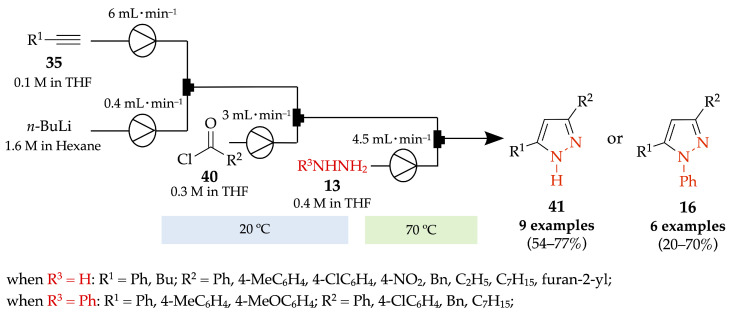
Transition metal-free continuous-flow process for the synthesis of 3,5-di- and 1,3,5-trisubstituted pyrazoles **41** and **16**, respectively.

A transition metal-free continuous-flow process for the synthesis of 3,5-di- and 1,3,5-trisubstituted pyrazoles **41** and **16**, respectively, was described by Kandasamy et al. The reaction of terminal aryl alkynes **35** with *n*-BuLi followed by coupling with acyl chloride derivatives **40** generated ynones, in situ, which then reacted with hydrazine derivatives **13** [47]. Nine *N*-unsubstituted pyrazoles **41** were obtained with moderate to good yields (54–77%) while six *N*-phenyl derivatives **16** were achieved with low to good yields (20–70%), with a total residence time around 70 min and under mild reaction conditions (Scheme 11).

Pyrazoles and related derivatives, such as pyrazolines, can also be obtained starting from less conventional substrates such as tetrazoles or pyridines.

**Scheme 12:**
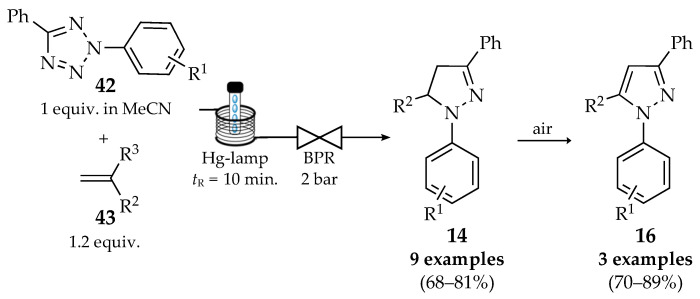
Photochemical synthesis of pyrazolines **14** and pyrazoles **16** from tetrazoles in flow conditions.

The synthesis starting from tetrazoles is not a direct or standard transformation but can be achieved through specific strategies involving suitable chemical intermediates and transformations. For example, a continuous photochemical click reaction was reported by Burke et al., which consists of the in situ generation of nitrile imine dipoles from aryl tetrazoles **42** and reaction with dipolarophiles **43**, affording diverse pyrazolines **14** and pyrazoles **16** (Scheme 12) [48]. Nine pyrazoline derivatives **14** were obtained with moderate to good yields (68–81%), and three of them were oxidized, affording the corresponding pyrazoles **16** with good yields (70–89%) [48,49]. Contrarily to UV-A LEDs, Hg-lamps with a filter allowed for achieving the expected products through photolysis of the nitrile imine intermediates. This flow process ensures a safe release of the nitrogen gas formed during the reaction by using a backpressure regulator and can generate gram quantities of products.

**Scheme 13:**
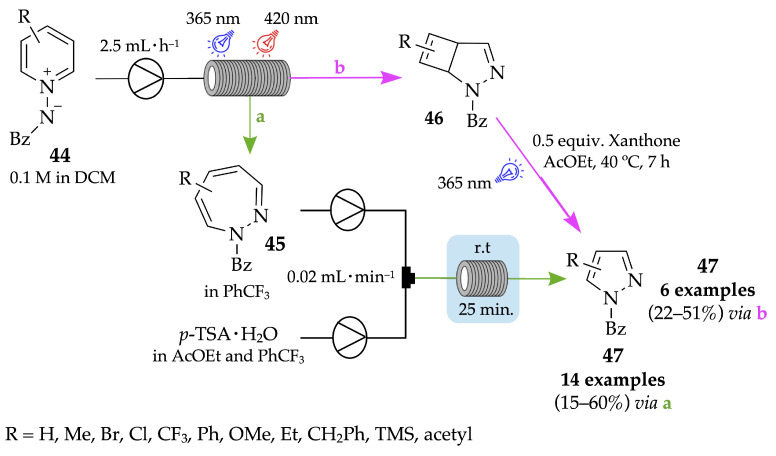
Expansion and/or rearrangement and cyclization of pyridinium salts and subsequent carbon deletion for the synthesis of pyrazoles **47**, under flow conditions.

The synthesis of pyrazoles from pyridines involves strategic transformations of the pyridine ring to form the characteristic five-membered heterocyclic ring of pyrazoles. This typically requires functional group modifications and ring contraction, which can be achieved through various chemical methods. Recently, Luo and colleagues developed a convenient and efficient two-step flow process for the late-stage modification of complex pyridine-containing drugs through an expansion and/or rearrangement cyclization of pyridinium salts **44** and subsequent carbon deletion [50]. As a representative example, a solution of benzoyl(pyridin-1-ium-1-yl)amide **44** in DCM was pumped into a photochemical reactor equipped with two 365 nm LEDs for 4 h, at room temperature and under nitrogen atmosphere. After purification by column chromatography, the corresponding 1,2-diazepine **45** was obtained with 82% yield. Then, a solution of the diazepine intermediate **45** in benzotrifluoride (PhCF_3_) was pumped through a fluorinated ethylene-propylene (FEP) capillary tube, at the same time as a solution of *para*-toluenesulfonic acid (*p*-TSA) in ethyl acetate and PhCF_3_, for 25 min. Finally, pure PhCF_3_ was loaded into the syringe to help push the mixture all the way through the reactor. Phenyl(1*H*-pyrazol-1-yl)methanone derivatives **47** were achieved in low-to-moderate yields (15–60%) through a carbon(2,3 or 5,6) deletion (via **a**). On the other hand, a rearrangement and cyclization also occurred when pyridinium salts were exposed to a 365 nm LED followed by a 420 nm LED, leading to the formation of the corresponding bicyclic pyrazoline products **46**. However, in this case, phenyl(1*H*-pyrazol-1-yl)methanones **47** were achieved in batch conditions, through a carbon(3,4 or 4,5) deletion promoted by irradiation with a 365 nm LED (via **b**) (Scheme 13). It is important to highlight that the absence or presence and the position of the substituent (R) of each obtained pyrazole derivative depends on the type of carbon deletion reaction.

**Scheme 14:**
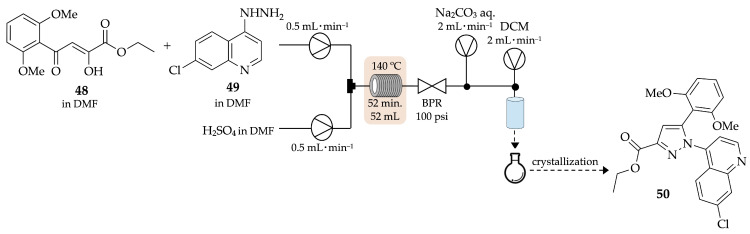
Continuous-flow synthesis of pyrazole **50** based on the Knorr cyclocondensation.

In 2013, Battilocchio and his group performed a continuous-flow reaction based on the Knorr cyclocondensation of compounds **48** and **49**, in DMF, to afford ethyl 1-(7-chloroquinolin-4-yl)-5-(2,6-dimethoxyphenyl)-1*H*-pyrazole-3-carboxylate **50** (Scheme 14) [51]. Although the yield was similar to the one achieved under batch conditions, the proposed flow method has shown to be highly efficient in the reaction’s workup step, requiring minimal user intervention due to the use of a semipermeable membrane that allows the passage of the organic phase whilst retaining the aqueous phase.

**Scheme 15:**
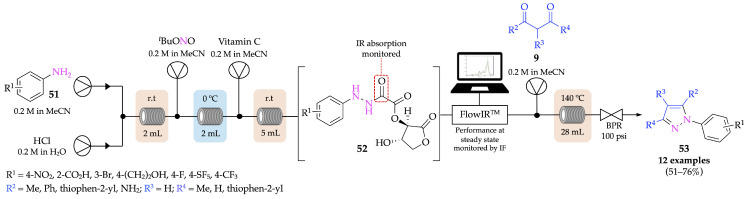
Four-stage multistep setup for continuous-flow production of pyrazoles **53** from anilines.

Poh et al. developed a four-step continuous flow setup for the conversion of anilines **51** into pyrazoles **53**, with 51–76% yields, where the diazotization step was performed in situ to avoid the isolation and exposure of hazardous, unstable and/or explosive intermediates (hydrazide and hydrazine derivatives), thus increasing safety. For the reduction stage, a more sustainable approach was applied by using reductants that can be washed away from the product through simple aqueous extraction—in this case, vitamin C (Scheme 15) [52]. Furthermore, this flow procedure allows the screening of reaction conditions with minimal operator input, another major advantage over batch procedures. A large-scale reaction was attempted for one derivative (R^1^ = 4-NO_2_, R^2^ = thiophen-2-yl), affording 3.65 g of product with 40% yield.

**Scheme 16:**
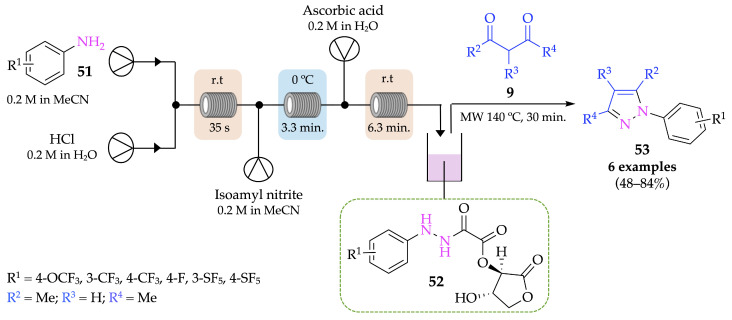
Continuous-flow–microwave hybrid approach for the synthesis of pyrazoles **53**.

A range of similar *N*-aryl pyrazoles **53** were successfully synthesized by Groves and colleagues, using a continuous-flow–microwave hybrid approach, based on the previous experiments reported by Poh et al. [53]. The diazotization of aniline derivatives **51** was performed with HCl and isoamyl nitrite instead of *^t^*BuONO, and ascorbic acid was used instead of vitamin C for the reduction step. While these first two steps were performed in flow conditions, the cyclocondensation step between oxamyl-hydrazide derivatives **52** and 1,3-dicarbonyl compounds **9** occurred in microwave at 140 °C for 30 min (Scheme 16). Results have shown that some derivatives were obtained in higher yields in flow (48–84%) compared to batch conditions (40–74%), while others presented similar yields in both methods. Despite this, in general, the flow process is preferable, especially regarding safety issues since it does not require the handling of hazardous intermediates.

**Scheme 17:**
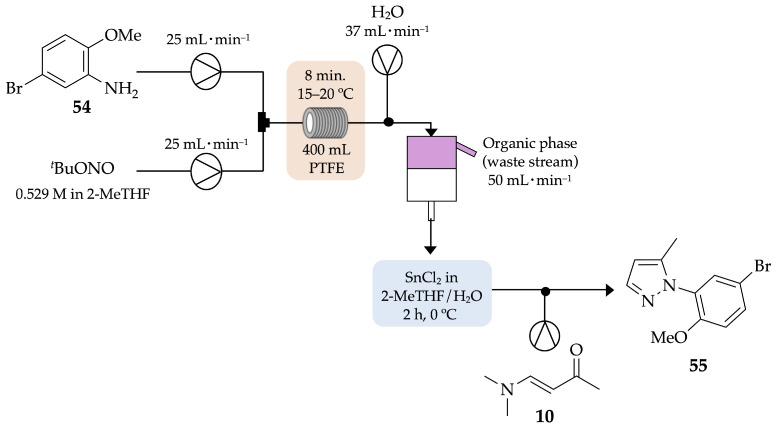
Continuous-flow synthesis of *N*-aryl-5-methylpyrazole **55**.

In 2012, Li and co-workers developed a continuous-flow process for the synthesis of *N*-aryl-5-methylpyrazole **55**, in safer conditions, by decreasing the exposure to diazonium salts and, consequently, the amount of nitrogen released after decomposition of the salts [54]. Thus, the diazotization of 5-bromo-2-methoxyaniline **54** followed by its reduction generated the hydrazine derivative, which then reacted with (*E*)-4-(dimethylamino)but-3-en-2-one **10**, based on the well-known Knorr cyclocondensation, to afford the corresponding pyrazole **55** with 51% yield, after 8 min of residence time (Scheme 17). When performed on a larger scale (~1 kg), the flow process proved its value once again since only 99 mmol of diazonium salts were produced, in comparison to the 4.9 mol produced in batch conditions.

**Scheme 18:**
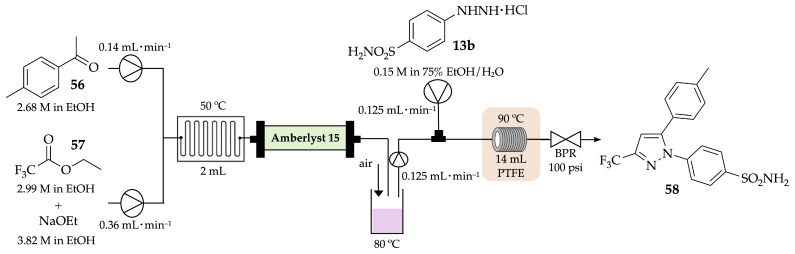
Continuous-flow process for the synthesis of celecoxib **58**.

Scholtz and Riley have improved the synthesis of celecoxib **58** using modern manufacturing technologies—in this case, flow chemistry [55]. Not only did they refine the batch conditions and obtain this anti-inflammatory drug with 90% yield but they also developed a continuous-flow approach where the same drug was achieved in the same yield on gram-scale with a residence time of 64 min, much less than the 20 h required in batch conditions (Scheme 18). Furthermore, the telescoped process was performed in greener conditions, due to the use of ethanol, and did not require the isolation or handling of intermediates.

**Scheme 19:**
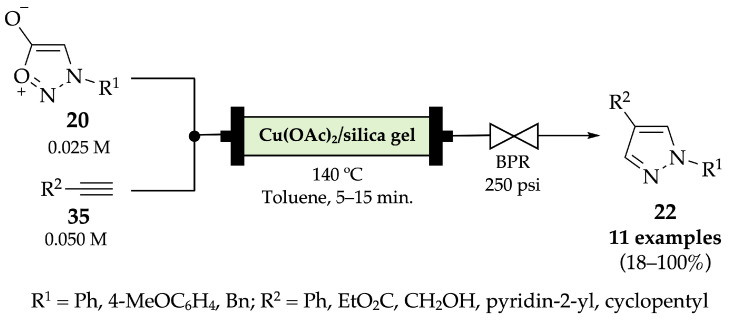
Copper-catalyzed cycloaddition of sydnones with terminal alkynes to produce pyrazoles **22** under flow conditions.

The synthesis of 1,4-disubstituted pyrazoles **22** from sydnones **20** and terminal alkynes **35** using silica-supported copper catalysts under flow conditions was reported by Comas-Barceló et al. in 2016 (Scheme 19) [56]. The described cycloaddition reaction was first carried out in batch conditions (21–100% yields) and then applied in continuous flow conditions (18–100% yields). The broad range of yields obtained is due to the distinct groups and substitution patterns of the alkyne counterpart. For example, the use of cyclopentylacetylene and propargyl alcohol derivatives decreased the reaction rates, thus requiring higher residence times (t*_R_* = 10–15 min instead of 5 min) to increase conversion. Even though both methods were able to afford the expected products in very similar yields, reaction time was significantly reduced from 2–20 h in batch to 5–15 min in continuous flow conditions. Furthermore, replacing *o*-dichlorobenzene by toluene in the presence of a back pressure regulator (BPR) improved the conversion ratio of the starting materials.

**Scheme 20:**
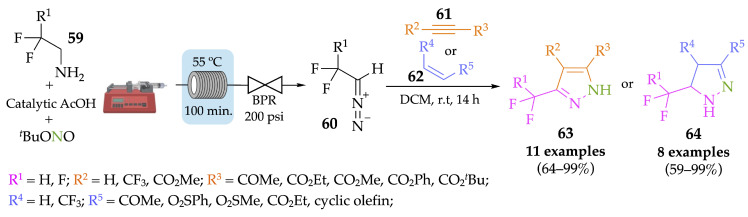
Synthesis of diazoalkanes in flow followed by cycloaddition in batch conditions to afford pyrazoles **63** and pyrazolines **64**.

Mertens et al. successfully produced a library of fluorinated diazoalkanes **60** from amines **59**, on a self-made microreactor, with the aim to use these intermediates to achieve fluorine-substituted pyrazoles **63** or pyrazolines **64** [57]. Thus, a [2 + 3] cycloaddition was performed between the synthesized diazo compounds and different dipolarophiles. Eleven pyrazole derivatives **63** were achieved in moderate to excellent yields (64–99%) when alkynes **61** were used in the cycloaddition step, while the same reaction with alkenes **62** resulted in eight pyrazoline derivatives **64**, also in moderate to very good yields (59–99%). Despite the good yields, safety issues must be considered when dealing with diazo compounds. Furthermore, the cycloaddition step was performed in batch conditions in a round-bottom flask (Scheme 20).

**Scheme 21:**
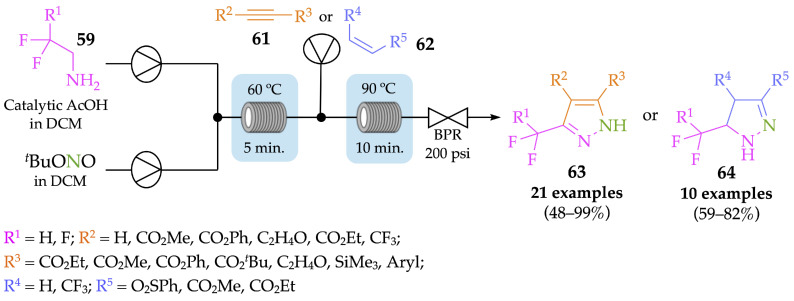
Continuous-flow setup for the in situ generation of diazo compounds and further cycloaddition with alkynes and alkenes to achieve pyrazoles **63** and pyrazolines **64**, respectively.

In order to mitigate the abovementioned issues, Britton and Jamison described an improved methodology, where both the in situ formation of diazoalkanes from amines **59** and their subsequent consumption in a cycloaddition with alkynes **61** and alkenes **62** occurred in flow conditions [58]. Apart from being a safer method, this new strategy significantly decreased the total reaction time from 16 h to 30 min. Pyrazole derivatives **63** were achieved in moderate to excellent yields (48–99%) when alkynes **61** were used in the cycloaddition step, while the same reaction with alkenes **62** resulted in pyrazoline derivatives **64**, in moderate to very good yields (59–82%) (Scheme 21). It is noteworthy that higher temperature and residence time were needed to promote the cycloaddition reaction with aryl alkynes.

### 4.2. Flow Synthesis of Pyrazole-Fused Scaffolds

#### 4.2.1. Indazoles

**Scheme 22:**
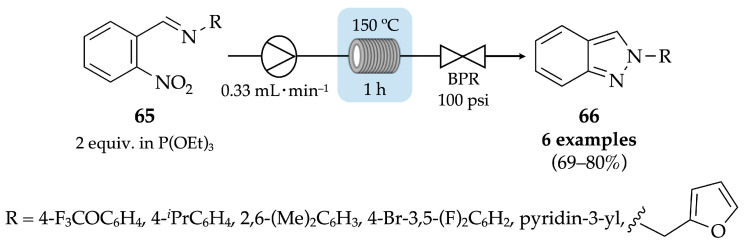
Thermal synthesis of *N*-arylindazoles **66** from nitroaromatic imines via Cadogan reaction, in flow conditions.

**Scheme 23:**
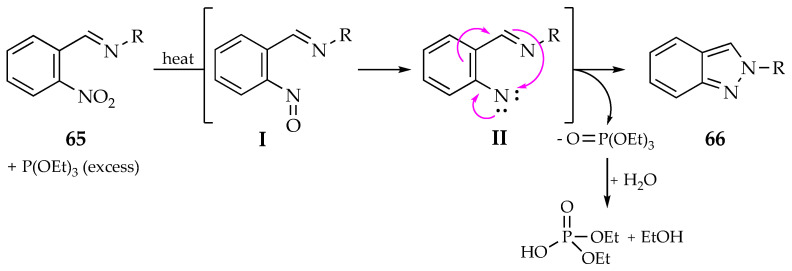
Cadogan reaction mechanism for the synthesis of *N*-substituted indazoles.

Duffy et al. reported the thermal synthesis of *N*-substituted indazoles from nitroaromatic imines **65** via the Cadogan reaction, both in batch and flow conditions. For the continuous-flow reaction, a solution of nitroaromatic imine substrate **65** in triethyl phosphite was pumped through two coiled reactors in series, at 150 °C, for 1 h. After purification by column chromatography, *N*-aryl indazoles **66** were achieved with 69–80% yield (Scheme 22) [59]. An exhaustive deoxygenation of the nitroaromatic imine **65** promoted by the phosphite to generate a nitrene (**II**), after the nitroso intermediate (**I**) formation, followed by an intramolecular cyclization process, seems to be the operating mechanism of this reductive cyclization to produce the *N*-aryl indazoles **66** (Scheme 23). Furthermore, a gram-scale reaction was also attempted, affording 4.2 g of compound **66** (R = 4-F_3_COC_6_H_4_) with 71% yield. Thus, the flow approach has proven to be more efficient considering that the expected indazole derivatives were obtained in higher yields and faster than in batch conditions. Nevertheless, it should be highlighted that a seventh derivative (when R = indazole) was produced only in batch conditions due to solubility issues.

**Scheme 24:**
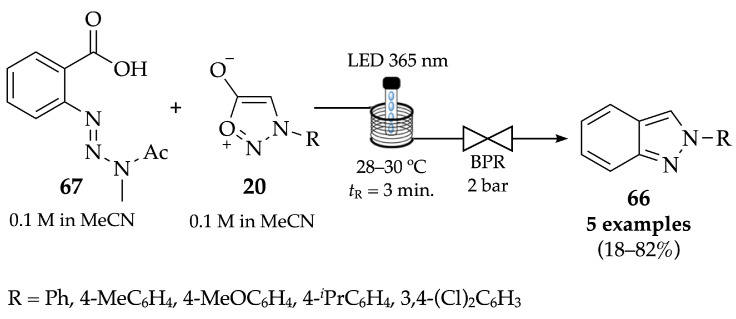
Continuous-flow synthesis of *N*-arylindazoles **66** by photochemical generation of benzynes and subsequent trapping with sydnones.

Bracken and co-workers described a photochemical methodology for the generation of benzynes from precursors and their subsequent trapping with sydnones to achieve indazoles. A solution of benzyne precursor **67** and sydnone derivatives **20** in acetonitrile was passed through a 10 mL coil of a photoflow reactor with an LED emitting at 365 nm, at constant temperature and pressure (Scheme 24) [60]. *N*-Arylindazoles **66** were obtained in moderate to good yields (70–82%), except for the chlorine derivative, which was obtained with only 18% yield. This might be due to the fact that 3,4-dichlorophenyl substituent on the sydnone counterpart is an electron withdrawing group (EWG), which supports the results Fang and colleagues obtained in 2011, since no product was isolated when using EWG-substituted sydnones, such as the 4-nitrophenyl-substituted ones [61]. Furthermore, when a proline-derived sydnone was used, 2,3-dihydro-1*H*-pyrrolo[1,2-*b*]indazole was achieved with 68% yield. Moreover, the 2-phenyl-2*H*-indazole was achieved with 68% yield, when scaling up the reaction from 0.1 mmol to 4.1 mmol.

**Scheme 25:**
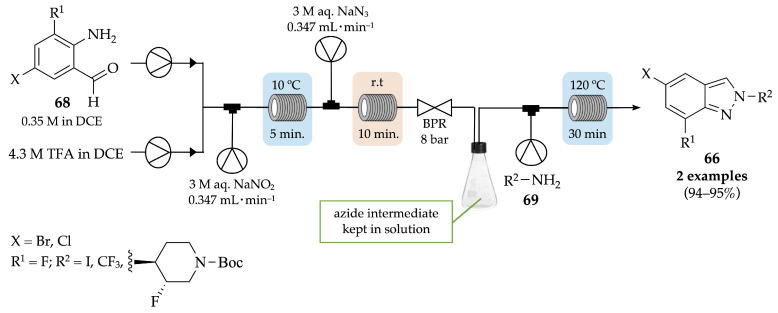
Three-step-flow procedure for the synthesis of indazole derivatives **66**.

To help reduce and limit the risks associated with the handling of large amounts of hazardous reagents and intermediates in the batch mode, Lehmann et al. described an alternative three-step-flow procedure to achieve indazole derivatives that are crucial precursors for the synthesis of highly potent and selective toll-like receptor (TLR) 7 and 8 antagonists (Scheme 25) [62]. The two-step formation of unstable azides was performed in flow, starting from the reaction of *o*-aminobenzaldehyde derivatives **68** and TFA with sodium nitrite, to produce the corresponding diazonium salts, which then reacted with sodium azide. After quenching with an aqueous solution of potassium carbonate, an azide solution was obtained and used for the next step, without further purification. A mixture of azide solution and amine derivative **69** in toluene was pumped through a copper coil at 120 °C, with a residence time of 30 min. Indazole derivatives **66** were obtained with up to 95% yield (crude), on a scale of 200 g, and further used as key intermediates for the synthesis of TLR7 and TLR8 antagonists.

This approach not only avoided the isolation of hazardous azides but also provided higher purity and yield for the two critical steps—azide formation and thermal cyclization.

#### 4.2.2. Pyrazolopyrimidinones

**Scheme 26:**
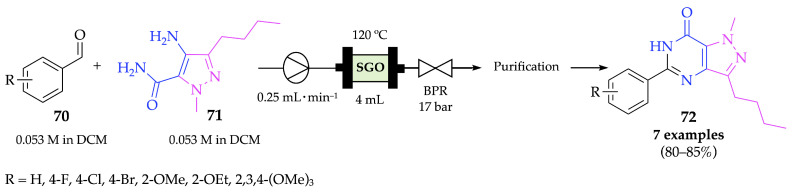
Synthesis of pyrazolopyrimidinones **72** under flow conditions.

Pyrazolopyrimidinone is a major scaffold of several drugs, widely used in the pharmaceutical field due to its remarkable biological properties, which is the reason why this example was also included in the present review. This type of compound has not only been demonstrated to have anti-inflammatory, anticancer, antimicrobial, antidiabetic and neuroprotective activities, among others, but also to be a phosphodiesterase 5 (PDE-5) inhibitor [63,64,65,66,67,68]. In fact, the pyrazolopyrimidinone scaffold is present in the structure of the well-known sildenafil (Viagra^®^) [69,70]. Thus, Sthalam and his group described a flow process for the synthesis of this fundamental drug-core, starting from arylaldehydes **70** and pyrazoles **71** [71]. A solution of the starting materials in DCM was pumped through a sulphonated graphene oxide catalyst at 120 °C (Scheme 26). After purification by flash column chromatography, pyrazolopyrimidinone derivatives **72** were obtained in very good yields (80–85%). Although the batch synthesis of these derivatives was achieved with similar yields, the reaction time was significantly reduced from 9 h to 16 min, using flow conditions. The described flow process allowed the synthesis of sildenafil on a large scale of up to 5.7 g per day.

#### 4.2.3. Pyrazolopyridines

The pyrazole ring can be fused with a pyridine ring to form the pyrazolopyridine scaffold, which also plays a key role in therapeutics. Recently, Donaire-Arias et al. published a review on the synthetic approaches and biomedical applications of pyrazolopyridines, highlighting their antitumoral and anti-inflammatory potential for the treatment of nervous system diseases [72].

**Scheme 27:**
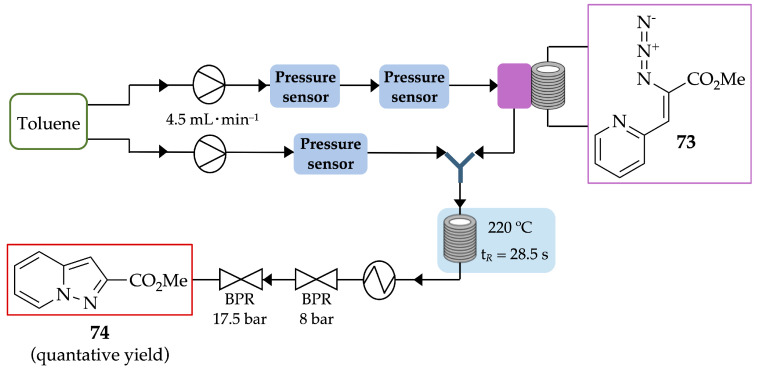
Synthesis of pyrazolo[1,5-*a*]pyridine **74** by thermolysis of azidoacrylates, under flow conditions.

O’Brien et al. demonstrated a simple, fast and efficient procedure for the thermolysis of azidoacrylates **73** in continuous flow to achieve a vast range of heterocycles, such as pyrazolo[1,5-*a*]pyridine **74** [73]. The azide counterpart **73** was injected through a PFA loop, while the solvent was pumped. The reaction occurred in a coil reactor at 220 °C with a residence time of 28.5 s. Methyl pyrazolo[1,5-*a*]pyridine-2-carboxylate **74** was obtained in quantitative yield, without further purification (Scheme 27), in both milligram and gram scales.

**Scheme 28:**
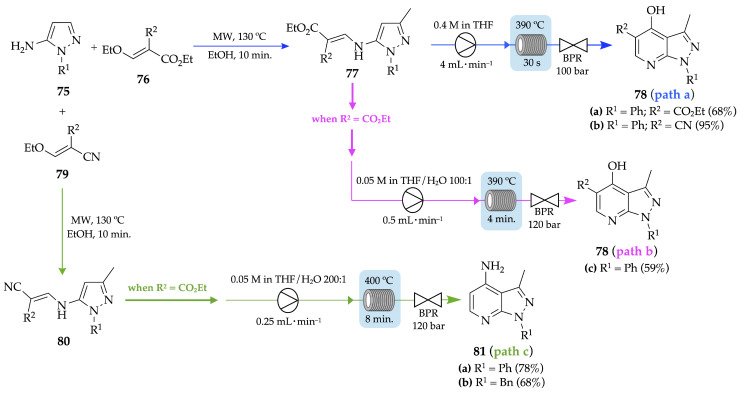
Microwave–flow hybrid approach for the synthesis of pyrazolo[3,4-*b*]pyridin-4-ols **78a**–**c** and pyrazolo[3,4-*b*]pyridin-4-amines **81a**,**b**.

A microwave–flow hybrid approach for the synthesis of pyrazolo[3,4-*b*]pyridine derivatives **78** and **81** based on Gould–Jacobs-type reactions was reported by Tsoung and her group, in 2016 [74]. A Michael-addition of 5-aminopyrazole **75**, and alkoxymethylene derivatives **76** or **79**, followed by elimination of the allylic ethoxy-group gave the corresponding aminomethylene intermediates **77** or **80**, respectively. The reaction was performed in the microwave, in ethanol, at 130 °C, and the intermediates were obtained as solids after precipitation. Pyrazolo[3,4-*b*]pyridin-4-ols **78a** and **78b** were obtained in moderate (68%) and excellent yields (95%), respectively, by thermal cyclization of intermediates **77**, under flow conditions (390 °C, t*_R_* = 30 s, 100 bar) (Scheme 28, path a), on a millimolar scale. It was also uncovered that slight changes in the flow setup or conditions could lead to different pyrazolo[3,4-*b*]pyridines. Thus, an increase in pressure from 100 to 120 bar and employment of a mixture of THF/H_2_O 100:1 as solvent instead of 100% THF promoted not only thermal cyclization of the intermediate **77a** (R^2^ = CO_2_Et), but also decarboxylation, thus affording decarboxylated pyrazolo[3,4-*b*]pyridin-4-ol **78c** (t*_R_* = 4 min) (Scheme 28, path b). On the other hand, pyrazolo[3,4-*b*]pyridin-4-amines **81a** and **81b** were achieved with 78% and 68% yields, respectively, by thermal cyclization of intermediate **80** and decarboxylation, under flow conditions (400 °C, t*_R_* = 8 min, 120 bar) (Scheme 28, path c).

## 5. Challenges and Future Directions

While flow chemistry offers numerous advantages, challenges remain concerning the reactors’ design to ensure that diffusion rates do not limit reaction rates, and especially the development of specialized reactors for heterogeneous reactions and solid-phase synthesis. Another difficulty still to be overcome is the management of side reactions in multi-component systems. In-line process analysis can be particularly important in these reactions for real-time monitoring of reaction parameters and products formed. Also, downstream processes—namely, streamlining separation, purification and isolation steps—must be seamlessly integrated with flow reactions. Techniques such as membrane filtration, crystallization and liquid–liquid extraction need to be optimized for continuous operation. Future directions in this research area comprise reaction automatization and the development of better modular flow systems for multi-step synthesis of complex heterocycles.

Despite its advantages, flow chemistry still faces economic barriers that hinder its widespread adoption. These include the high initial investment cost, due to the need for specialized equipment (flow reactors, pumps, microreactors and automation systems) that are expensive compared to traditional batch reactors, and flow implementation may require modifications in the existing laboratory or industrial setups. Also, reaction conditions (temperature, pressure and residence time) must be carefully optimized, which can be costly. The integration of flow reactors with existing systems, namely, batch systems, may be complex and expensive. On the other hand, flow systems require periodic cleaning due to reactor fouling and clogging; frequent maintenance; and replacement of components like tubing, pump and reactors, thus increasing costs. These costs and technical issues may explain the existing reluctance in shifting from batch to flow. To expand the implementation of flow chemistry, training future generations of chemists and engineers to operate flow chemistry systems is also essential as it demands specialized knowledge in fluid dynamics, automation and reactor design.

## 6. Conclusions

The interest in the pivotal heterocyclic compounds reported in this review—pyrazoles, pyrazolines and fused pyrazoles—has been growing owing to their relevance in the pharmaceutical, agrochemical and materials science fields. However, the conventional methods for their synthesis present some obstacles regarding selectivity, scalability and safety. Flow chemistry has revolutionized the synthesis of five-membered nitrogen-containing heterocycles. The examples discussed in this monography showed that the application of flow chemistry in the synthesis of pyrazoles, pyrazolines and indazoles was particularly important to address limitations of traditional methods enabling the development of more efficient, scalable and environmentally friendly processes. As advancements in reactor technology and process integration continue, the role of flow chemistry in the synthesis of these important heterocycles is expected to expand further, paving the way for innovative applications in pharmaceuticals and materials development.

## Data Availability

Data sharing is not applicable.

## Abbreviations

The following abbreviations are used in this manuscript:

BPR	Back pressure regulator
Bn	Benzyl
Bu	Butyl
Bz	Benzoyl
cat	Catalyst
DCE	1,2-Dichloroethane
DCM	Dichloromethane
DIEA	*N*,*N*-Diisopropylethylamine
DMF	Dimethylformamide
DMSO	Dimethylsulfoxide
equiv	Molar equivalent
Et	Ethyl
EVI2	Ethyl viologen diiodide
EWG	Electron withdrawing group
FEP	Fluorinated ethylene-propylene
^1^H NMR	Proton nuclear magnetic resonance spectroscopy
*^i^*Bu	*iso*-Butyl
*^i^*Pr	*iso*-Propyl
IR	Infrared
LED	Light emitting diode
Me	Methyl
MW	Microwave
NMP	*N*-Methyl-2-pyrrolidone
PDE-5	Phosphodiesterase 5
PFA	Perfluoroalkoxy
Ph	Phenyl
PTFE	Polytetrafluoroethylene
*p*-TSA	*para*-Toluenesulfonic acid
r.t	Room temperature
SGO	Sulfonated graphene oxide
*^i^*Bu	*t*-Butyl
TFA	Trifluoroacetic acid
THF	Tetrahydrofuran
TLR	Toll-like receptor
TMEDA	*N*,*N*,*N’*,*N’*-Tetramethylethylenediamine
TMS	Tetramethylsilane
t*_R_*	Residence time
